# ‘When Birds of a Feather Flock Together’: Synesthetic Correspondences Modulate Audiovisual Integration in Non-Synesthetes

**DOI:** 10.1371/journal.pone.0005664

**Published:** 2009-05-27

**Authors:** Cesare Valerio Parise, Charles Spence

**Affiliations:** Crossmodal Research Laboratory, Department of Experimental Psychology, University of Oxford, Oxford, United Kingdom; Istituto di Neurofisiologia, Italy

## Abstract

**Background:**

Synesthesia is a condition in which the stimulation of one sense elicits an additional experience, often in a different (i.e., unstimulated) sense. Although only a small proportion of the population is synesthetic, there is growing evidence to suggest that neurocognitively-normal individuals also experience some form of synesthetic association between the stimuli presented to different sensory modalities (i.e., between auditory pitch and visual size, where lower frequency tones are associated with large objects and higher frequency tones with small objects). While previous research has highlighted crossmodal interactions between synesthetically corresponding dimensions, the possible role of synesthetic associations in multisensory integration has not been considered previously.

**Methodology:**

Here we investigate the effects of synesthetic associations by presenting pairs of asynchronous or spatially discrepant visual and auditory stimuli that were either synesthetically matched or mismatched. In a series of three psychophysical experiments, participants reported the relative temporal order of presentation or the relative spatial locations of the two stimuli.

**Principal Findings:**

The reliability of non-synesthetic participants' estimates of both audiovisual temporal asynchrony and spatial discrepancy were lower for pairs of synesthetically matched as compared to synesthetically mismatched audiovisual stimuli.

**Conclusions:**

Recent studies of multisensory integration have shown that the reduced reliability of perceptual estimates regarding intersensory conflicts constitutes the marker of a stronger coupling between the unisensory signals. Our results therefore indicate a stronger coupling of synesthetically matched vs. mismatched stimuli and provide the first psychophysical evidence that synesthetic congruency can promote multisensory integration. Synesthetic crossmodal correspondences therefore appear to play a crucial (if unacknowledged) role in the multisensory integration of auditory and visual information.

## Introduction

Over the last few decades, studies have shown that the behavior of non-synesthetic individuals is affected by multisensory interactions that have traditionally been regarded as the prerogative of the synesthetic population [1]–[11]. A paradigmatic example of this is the synesthetic correspondence between auditory pitch and visual size, whereby higher-pitched tones are associated with smaller objects and lower-pitched tones with larger objects [1], [8]–[10], [12]. Synesthetic associations in neurocognitively-normal individuals have typically been studied by means of the speeded classification paradigm, in which participants have to classify a series of stimuli in one sensory modality while trying to ignore concurrent task-irrelevant stimuli presented in a second modality [2], [13]. The classic finding is that when the irrelevant stimulus is congruent with the relevant one (i.e., when a high pitched tone is presented with a small visual object), participants respond more rapidly and accurately than on incongruent trials, where the relevant and irrelevant stimuli do not match synesthetically [2], [3], [13]. Despite a growing number of studies showing synesthetically driven interactions between crossmodal stimuli, there is to date no psychophysical evidence that synesthetic congruency actually modulates multisensory integration.

Here we investigate the role of synesthetic correspondences on the integration of pairs of temporally (Experiment 1 and 2) or spatially (Experiment 3) conflicting auditory and visual stimuli. When spatiotemporally conflicting stimuli from different modalities are integrated, small conflicts are often compensated for, giving rise to the ventriloquist effect, whereby the conflicting stimuli are perceptually “pulled” together toward a single spatiotemporal onset [14]–[17]. Participants therefore tend to perceive combinations of spatiotemporally conflicting stimuli as unitary multisensory events and become less sensitive to any crossmodal conflicts that may be present [18]. Multisensory integration, in fact, has the cost of hampering the brain's access to the individual sensory components feeding into the integrated percept, thus reducing the reliability of estimates of potential crossmodal conflicts [19], [20]. Reliability is defined here as the inverse of the squared discrimination threshold, the just noticeable difference (JND), that is the minimal difference along a given dimension between a test and a standard stimulus that an observer can detect at a specified level above chance.

According to Bayesian models of multisensory integration, the reliability of participants' estimates regarding intersensory conflicts is proportional to the strength of coupling between the integrated signals [21]. In particular, strong coupling may lead to a complete fusion of the original signals into the integrated percept that is evidenced behaviorally by a reduction in the reliability of conflict estimates (i.e., higher discrimination thresholds), whereas a weaker coupling only leads to partial fusion, with the system still retaining access to reliable conflict estimates (i.e., lower discrimination thresholds). The strength of coupling is a function of the sensory system's prior knowledge that the crossmodal stimuli “go together”: such prior knowledge about the mapping between signals has been modeled by a coupling prior [19], representing the expected (i.e., a priori) joint distribution of the signals. The coupling prior influences the strength of coupling in inverse proportion to its variance: A variance approaching infinity (i.e., a flat prior) means that the signals are treated as independent and there is no interaction between the signals presented in the different modalities; conversely a variance approaching 0 indicates that the signals are completely fused into the integrated percept, whereas intermediate values determine a coupling of the signals without sensory fusion. The variance of the coupling prior (and therefore the strength of coupling), in turn, is known to be determined by the previous knowledge that the stimuli originate from a single object [22] or event [23] and by a repeated exposure to statistical co-occurrence of the signals [21].

Within such a framework, if synesthetic information is used by the perceptual system to integrate stimuli from different modalities, the strength of coupling should be higher for synesthetically congruent combinations of stimuli as compared to synesthetically incongruent combinations. Therefore, when presented with synesthetically congruent audiovisual stimuli that are either asynchronous or spatially discrepant, participants' estimates requiring access to such conflicts, such as judgments regarding the relative temporal order or the relative spatial location of the stimuli, should be less reliable (i.e., higher discrimination thresholds for spatiotemporal conflicts) as compared to conditions in which the conflicting stimuli are synesthetically incongruent.

A similar effect has recently been reported in the temporal domain with audiovisual speech stimuli (human voices and moving lips) presented asynchronously that were either matched (i.e., voices and moving lips belonging to the same person) or mismatched (i.e., voices and moving lips belonging to a different person). When both modalities provide congruent information, more pronounced multisensory integration takes place, leading to a “unity effect”, which is evidenced behaviorally by an increase of the discrimination thresholds for audiovisual temporal asynchronies [24], [25]. Interestingly, subsequent studies have shown that the phenomenon disappears when participants are presented with realistic non-speech stimuli, thus suggesting that the “unity effect” might be specific to speech [24], [26].

An increase of the discrimination thresholds for spatial and temporal conflict when audiovisual stimuli are synesthetically matched would provide the first psychophysical evidence that synesthetic congruency promotes multisensory integration, thus qualifying synesthetic congruency as a novel, additional cue to multisensory integration. Moreover, such a result would constitute the first empirical demonstration that the “unity effect” is not a prerogative of speech stimuli and that it can also occur in the spatial domain. We anticipate that, in keeping with our predictions, participants' estimates regarding both spatial and temporal conflicts were less reliable with synesthetically congruent audiovisual stimuli than with synesthetically incongruent stimuli, thus supporting the claim that synesthetic congruency promotes multisensory integration.

## Materials and Methods

### Experiment 1: Temporal Conflict – Pitch-Size

Twelve non-synesthetic participants, with normal vision and audition, made unspeeded audiovisual temporal order judgments (TOJs) regarding which stimulus (i.e., visual or auditory) had been presented second [27]. Visual stimuli consisted of light grey circles presented for 26 ms at the centre of a CRT screen against a white background, and subtending 2.1° (small stimulus) or 5.2° (large stimulus) of visual angle at a viewing distance of 55 cm. The auditory stimuli consisted of 26 ms pure tones, with 5 ms linear ramps at on- and off-set and delivered via headphones against background white noise. The frequency of the tones was 300 Hz (low pitched) or 4500 Hz (high pitched). High and low pitched tones in this and the following experiments were made equally loud for each participant through an adaptive psychophysical procedure (QUEST, [28]).

**Figure 1:**
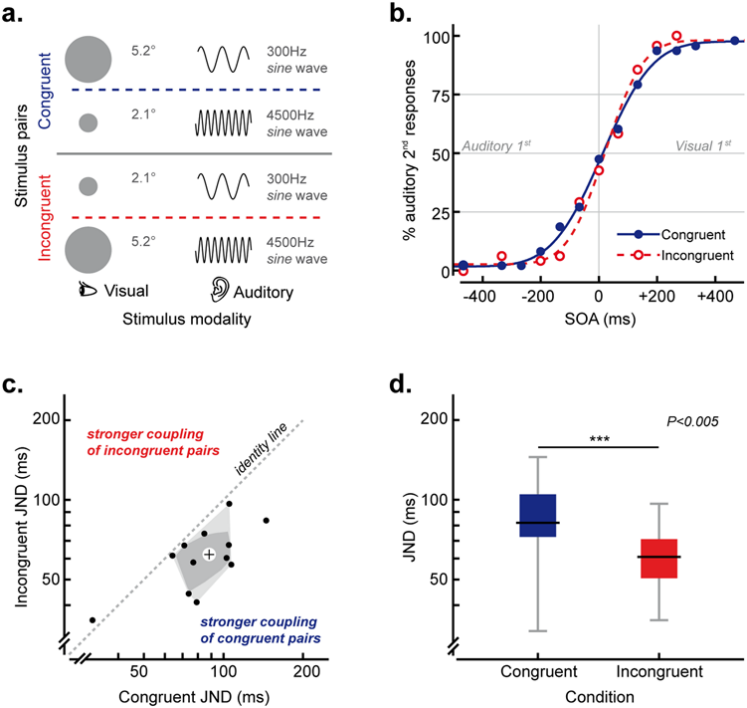
Experiment 1: stimuli and results. a. Pairs of auditory and visual stimuli presented in synesthetically congruent (top) and incongruent trials (bottom) in Experiment 1. b. Psychometric functions describing performance on synesthetically congruent (continuous line) and incongruent (dashed line) conditions in Experiment 1. Filled and empty circles represent the proportion of “auditory second” responses for each SOA tested averaged over all participants of Experiment 1. c. Scatter and bagplot [39] of participants' sensitivity (JNDs) on congruent vs. incongruent trials (log-log coordinates). Points below the identity line indicate a stronger coupling of congruent stimuli. The cross at the centre of the bag represents the depth median. d. Sensitivity of participants' responses (JNDs) on congruent and incongruent trials in log scale. The central lines in the boxes represent the median JND, the boxes indicate the first and third quartiles, and the whiskers, the range of the data.

A visual and an auditory stimulus were presented on each trial with a variable stimulus onset asynchrony (SOA; ±467, ±333, ±267, ±200, ±133, ±76 and 0 ms, negative values indicate that visual stimulus trailed the auditory stimulus, positive values indicate that visual stimulus led). Each SOA was presented 10 times (20 for the 0 ms SOA) in each condition (i.e., in both the synesthetically congruent and synesthetically incongruent conditions). The auditory and visual stimuli presented on each trial were equiprobably either synesthetically congruent along the above-mentioned pitch-size dimension (i.e., a higher-pitched tone was paired with a smaller visual stimulus or a lower-pitched tone was paired with a larger visual stimulus) or else synesthetically incongruent (i.e., a higher-pitched tone was paired with a larger visual stimulus and a lower-pitched tone was paired with a smaller visual stimulus, see Figure 1A). In order to maximize the alternation of congruent and incongruent trials, no more than 2 trials from the same condition were presented in a row. The participants had to perform an unspeeded discrimination task in which they had to indicate the modality of the second stimulus presented on each trial by pressing one of two response keys.

### Experiment 2: Temporal Conflict – Pitch/Waveform-Shape

**Figure 2:**
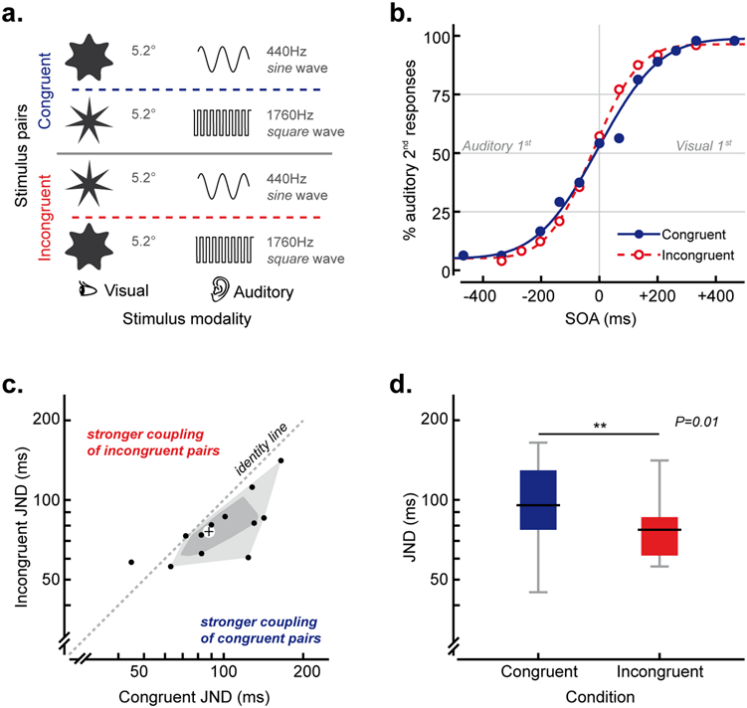
Experiment 2: stimuli and results. a. Pairs of auditory and visual stimuli presented in Experiment 2. b. Psychometric functions describing performance on synesthetically congruent (continuous line) and incongruent (dashed line) conditions in Experiment 2. c. Bagplot [39] of participants' sensitivity (JNDs) on congruent vs. incongruent trials. d. Participants' sensitivity (JNDs), on congruent and incongruent trials in Experiment 2.

The generalizability of the results of Experiment 1 was tested in a second experiment by varying the synesthetic correspondence between the auditory features of pitch and waveform and the visual features of curvilinearity and the magnitude of the angles of regular shapes (see [2], [31]; see Fig. 2A). The visual stimuli consisted of black 7-pointed stars presented for 26 ms against a white background and subtending 5.2° of visual angle. One star was curvilinear and had a ratio of inscribed to circumscribed circles of 0.65, whereas the other star was angular and had a ratio of inscribed to circumscribed circles of 0.55. The auditory stimuli, delivered via headphones against background white noise, consisted of 26 ms tones with 5 ms linear ramps at on- and off-set. One auditory stimulus consisted of a high pitched (1760 Hz), square waved tone, whereas the other had lower frequency (440 Hz) and sinusoidal wave.

The experimental procedure was the same as Experiment 1, with the exception that the compatible stimulus combination here consisted of the presentation of the pointed star together with the higher pitched tone and the curvilinear star with the lower pitched tone. Conversely the incompatible stimulus pairs consisted of the pointed star coupled with the lower pitched tone and the curvilinear star with the higher tone.

### Experiment 3: Spatial Conflict

Twelve non-synesthetic participants, with normal vision and audition, made unspeeded judgments as to whether an auditory stimulus was presented to either the left or the right of a visual stimulus.

**Figure 3:**
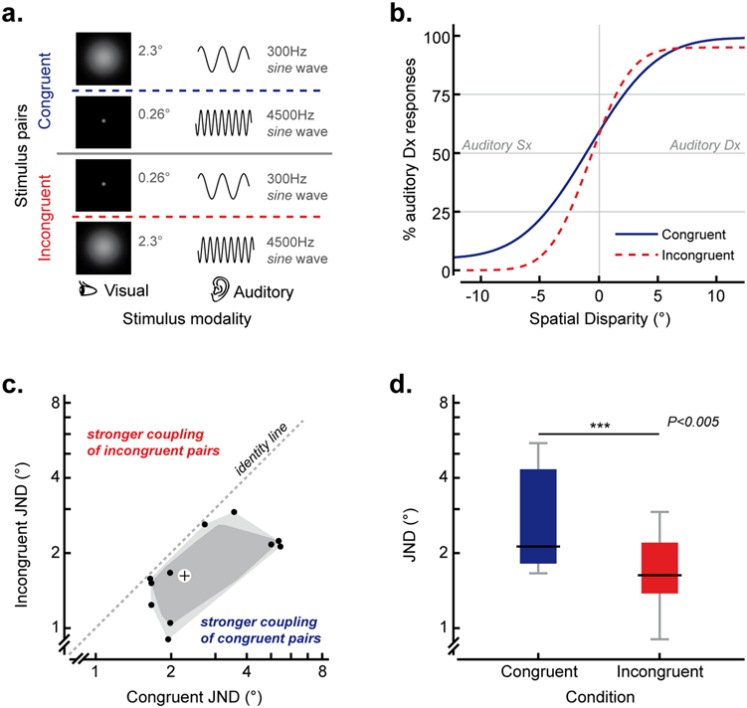
Experiment 3: stimuli and results. a. Pairs of auditory and visual stimuli presented in Experiment 3. b. Psychometric functions describing performance on synesthetically congruent (continuous line) and incongruent (dashed line) conditions in Experiment 3. c. Bagplot [39] of participants' sensitivity (JNDs) on congruent vs. incongruent trials. d. Participants' sensitivity (JNDs), on congruent and incongruent trials in Experiment 3.

The visual stimuli consisted of white Gaussian blobs projected for 200 ms against a black background on a fine fabric screen (width: 107.7 cm; height: 80.8 cm). The standard deviation of the Gaussian luminance profile of the blobs subtended 0.26° (small stimulus) or 2.3° (large stimulus) of visual angle at a viewing distance of 110.5 cm (a chinrest was used to control the head position). The auditory stimuli consisted of 200 ms pure tones with 5 ms linear ramps at on- and off-set; the frequency of the tones was 300 Hz (low pitched) or 4500 Hz (high pitched, see Fig. 3A). In order to provide richer spectral cues for auditory localization, the tones were convolved with white noise [32] and their intensity was modulated with a sinusoidal profile with a frequency of 50 Hz. The auditory stimuli were delivered from one of four loudspeaker placed behind the fabric screen (placed 5.2 cm and 15.6 cm to the left and the right of the midline of the screen) and their intensity was randomly jittered from trial to trial (between ±1% of the standard intensity) in order to avoid participants using any potential slight differences in the intensities of the sounds delivered by the 4 loudspeaker as auxiliary cues for sound source localization. White noise was delivered by an additional pair of loudspeaker placed behind the screen throughout the experimental session.

A train of 3 synchronous audiovisual events, with an interstimulus interval randomized between 150 ms and 300 ms, was presented on each trial with the source of the auditory stimulus randomly located to the left or the right of visual stimulus with the magnitude of the azimuthal displacement determined using an adaptive psychophysical procedure. At the beginning of the experiment we assumed a psychometric function fitted over a small set of hypothetical data points. In particular, we assumed that participants correctly responded “left” or “right” in 4 trials in which the auditory stimulus was placed 9.7° and 4.9° to the left or the right of the visual stimulus and fitted a cumulative Gaussian curve over these four points. Then, after each response, the curve was fitted again with the newly-collected data and the auditory stimulus that was presented on the next trial was randomly placed to the left or the right of the visual stimulus with a displacement normally randomized around 1 JND (s.d. 1 JND). This procedure was selected after preliminary results which indicated high variability in participants' ability to localize sounds, making it hard to preventively select an effective placement of the stimuli (as required the method of limits [33] and the method of constant stimuli [34]), and because it optimizes the information provided by each data by placing the stimuli around the regions that are more relevant to calculate the JND. In order to train participant to localize sounds, before running the experiment, they were required to perform a quick task (96 trials) where a sound was emitted by one of 8 loudspeakers placed behind the screen (4 to the left and 4 to the right of the vertical midline) and they had to determine whether it was coming from the left or the right of the screen's midline (visual feedback was provided after incorrect responses in the training block).

The auditory and visual stimuli presented on each trial were equiprobably either synesthetically congruent along the pitch-size dimension (i.e., a higher-pitched tone was paired with a smaller visual stimulus or a lower-pitched tone was paired with a larger visual stimulus) or else they were synesthetically incongruent (i.e., a higher-pitched tone was paired with a larger visual stimulus and a lower-pitched tone was paired with a smaller visual stimulus, see Fig. 3A). Two hundred and eighty trials were presented on each session (140 congruent and 140 incongruent). Participants performed an unspeeded discrimination task in which they had to press either the left or the right key of a computer mouse in order to indicate whether the auditory stimulus was coming from the left or the right of the visual stimulus.

### Ethics statement

This study was conducted in accordance to the declaration of Helsinki, and had ethical approval from the Department of Experimental Psychology at the University of Oxford. All participants provided written informed consent and received course credits or a £5 gift voucher in return.

## Results

### Experiment 1: Temporal Conflict – Pitch-Size

Separate psychometric functions for congruent and incongruent trials were calculated for each participant by fitting the ratios of “auditory second” responses plotted against SOAs with a cumulative Gaussian distribution [29] (see Fig. 1B). The just noticeable differences (JNDs), providing a measure of the reliability (i.e., the discrimination threshold) of participants' TOJs, were calculated for both synesthetically congruent and synesthetically incongruent conditions by subtracting the SOA at which participants made 75% “auditory second” responses from the SOA at which they made 25% “auditory second” responses and halving the result (see Fig. 1B–D). Synesthetic congruency had a significant influence on the reliability of participants' estimates (Wilcoxon Signed Rank Test Z = −2.903, p = .004), with smaller JNDs (indicating increased reliability) reported for synesthetically incongruent trials (median = 61 ms, interquartile range (IQR) = 72–104 ms) than for congruent trials (median = 82 ms, IQR = 51–71 ms). This result provides support for the claim that enhanced multisensory integration takes place for congruent as compared to incongruent audiovisual stimulus pairs. Eleven out of the 12 participants tested exhibited less reliable TOJ estimates for synesthetically congruent as compared to incongruent stimulus pairs (Sign Test, p = .006). Although the PSE data (denoting the point of maximum uncertainty in participants' judgments) do not provide relevant information regarding the strength of coupling (e.g., see [19], [30]) nor the “unity effect” (e.g., see [24]–[26]), statistical outcomes on the effect of synesthetic associations on the PSE are reported for completeness: Z = −0.549, p = .583.

### Experiment 2: Temporal Conflict – Pitch/Waveform-Shape

JNDs (calculated with the procedure described in Experiment1) were again significantly higher on the synesthetically congruent trials (median = 95 ms, IQR = 77–129 ms) than on the synesthetically incongruent trials (median = 77 ms, IQR = 61–86 ms, Wilcoxon-Test Z = −2.589, p = .010), with 10 out of 12 of the participants tested exhibiting higher discrimination thresholds in the congruent as compared to the incongruent condition (Sign Test, p = .039, see Fig. 2B–D). No significant effect of condition was found in the PSE data (Z = .893, p = .343).

### Experiment 3: Spatial Conflict

Separate psychometric functions were calculated for congruent and incongruent trials for each participant by fitting the ratios of “auditory right” responses plotted against spatial displacement (measured in degrees of visual angle, with negative values indicating that the auditory stimulus was placed to the left of the visual one) with a cumulative Gaussian distribution [29](see Figure 3B). Synesthetic congruency significantly influenced the reliability of participants' estimates (Wilcoxon Signed Rank Test Z = −3.059, p = .002), with smaller discrimination thresholds reported for synesthetically incongruent trials (mean = 1.7°, IQR = 0.9°) than for congruent trials (median = 2.2°, IQR = 2.6°), thus providing support for the claim that enhanced multisensory integration takes place for congruent as compared to incongruent pairs of audiovisual stimuli. All of the participants exhibited lower discrimination thresholds in response to spatial conflicts between synesthetically congruent as compared to incongruent stimulus pairs (Sign Test, p<.001, see Fig. 3C–D). Interestingly, synesthetic congruency also had a significant effect on the PSE data in this experiment: Z = −2.432, p = .015.

## Discussion

The results of the three experiments reported here demonstrate that synesthetic correspondences affect multisensory integration, as assessed by their effect on the reliability of participants' audiovisual TOJs and spatial localization judgments. In particular, estimates requiring access to temporal (Experiments 1 & 2) and spatial (Experiment 3) conflicts between synesthetically congruent auditory and visual stimuli were found to be less reliable (i.e., higher discrimination thresholds) than those requiring access to conflicts between synesthetically incongruent stimuli. A reduced reliability of the estimates requiring access to intersensory conflicts reflects the cost of multisensory integration and is the marker of a stronger coupling between the unisensory signals [19], [20], [30]. These results therefore indicate a stronger coupling of synesthetically congruent stimuli as compared to synesthetically incongruent stimuli and provide the first psychophysical evidence that synesthetic congruency can actually promote multisensory integration. It should be noted, however, that the synesthetic associations studied here (as well as in many other studies, see [1]–[3], [5], [7]–[10], [13]) are likely relative rather than absolute, depending on the particular range of stimuli used. What is called a ‘big’ circle, in fact, would most likely behave like a small circle if we happened to pair it with an even larger circle and the same argument would apply, *mutatis mutandis,* to any other potential stimulus features that happen to be considered (see [3] on this issue).

Considering that the unimodal signals used in our experiments were identical in both congruent and incongruent conditions (i.e., same signal reliability in both conditions), the difference in the strength of coupling reported here should be attributed to the knowledge of the participants' perceptual systems about which stimuli ‘belong together’ (or, rather, which normally co-occur) and should therefore be integrated. According to Bayesian integration models, such prior knowledge about stimulus mapping, the coupling prior, determines the strength of the coupling between the stimuli proportionally to its reliability (with reliability defined as the inverse of the squared variance of the coupling prior distribution), that is, the more the system is certain that two stimuli belong together (i.e., the smaller the variance of the coupling prior), the stronger such stimuli will be coupled [19], [30]. The effect of synesthetic associations in multisensory integration could, therefore, be interpreted in terms of differences in the variance of the coupling prior (i.e., smaller variance for synesthetically congruent stimulus pairs than for synesthetically incongruent pairs), that is to say that the synesthetic associations determine the strength of coupling by modulating the variance of the coupling prior distribution.

It should, however, be noted that our results might also be accounted for by the possibility that synesthetic associations modulate the tuning of multisensory spatio-temporal filters (see [35]). The early stages of sensory processing have, in fact, traditionally been modeled in terms of spatial and temporal filters operating upon the incoming sensory information (e.g. see [36], [37]). Their role, in a crossmodal setting, would be critical to determining the perceived temporal simultaneity and spatial coincidence of multisensory signals [35]. Synesthetic information might act on those filters by increasing their spatial and temporal constants under conditions of congruent crossmodal stimulation and by reducing such constants when the stimuli are incongruent. In keeping with the data reported here, a similar synesthetic modulation of the tuning of the multisensory spatio-temporal filters could also determine larger windows of both subjective simultaneity and spatial coincidence for congruent as compared to incongruent pairs of audiovisual stimuli.

The results of Experiments 1 and 2 also extend the finding of previous research on the “unity effect” by showing that an increase of the discrimination threshold for temporal asynchronies is not specific to matched audiovisual speech events: synesthetic congruency can also trigger robust unity effects. Vatakis and her colleagues [24]–[26] have conducted a number of studies on the integration of asynchronous but ecologically-valid audiovisual stimuli and consistently found that the “unity effect” is restricted to speech stimuli, thus concluding that speech is “special” inasmuch as the facilitatory effect on multisensory integration leading to the unity effect is specific to speech. Our results, therefore, not only extend the class of stimuli that are known to lead to a unity effect, but also suggest the hypothesis that synesthetic associations might also be “special” (or rather that audiovisual speech stimuli may not be so special, or unique, after all). In addition, the results of Experiment 3, showing that participants' discrimination thresholds for the spatial separation between auditory and visual stimuli are increased when the stimuli are synesthetically congruent, constitutes the first experimental evidence that the unity effect also occurs in the spatial domain, and thus provides additional evidence for the claim that the unity effect results from more pronounced multisensory integration.

While research has tended to focus on the spatiotemporal constraints of multisensory integration over the past 25 years [38], the results reported here demonstrate that synesthetic congruency provides an additional constraint on such processes.
